# Fever, hyperglycaemia and swallowing dysfunction management in acute stroke: A cluster randomised controlled trial of knowledge transfer

**DOI:** 10.1186/1748-5908-4-16

**Published:** 2009-03-16

**Authors:** Sandy Middleton, Christopher Levi, Jeanette Ward, Jeremy Grimshaw, Rhonda Griffiths, Catherine D'Este, Simeon Dale, N Wah Cheung, Clare Quinn, Malcolm Evans, Dominique Cadilhac

**Affiliations:** 1 St Vincents and Mater Health Sydney, Victoria St, Darlinghurst, 2010, NSW, Australia; 2 Hunter Stroke Service, Neurology Unit, John Hunter Hospital and Hunter Medical Research Institute, Lookout Rd, New Lambton Heights NSW 2305, Australia; 3 Department of Epidemiology and Community Medicine, University of Ottawa, 451 Smyth Road, Ottawa, Ontario K1H 8M5, Canada; 4 Canada Research Chair in Health Knowledge, Transfer and Uptake, Director, Clinical Epidemiology Program, Ottawa Health Research Institute, 1053 Carling Avenue, Administration Building, Room 2-017, Ottawa, Ontario K1Y 4E9, Canada; 5 School of Nursing and Midwifery, University of Western Sydney, Locked Bag 1797, Penrith South DC NSW 1797, Australia; 6 Centre for Clinical Epidemiology and Biostatistics, School of Medicine and Public Health, Faculty of Health, The University of Newcastle, University Drive, Callaghan, Newcastle NSW 2300, Australia; 7 National Centre for Clinical Outcomes Research (NaCCOR), Nursing and Midwifery, ACU National, PO Box 968, North Sydney, NSW 2059, Australia; 8 Department of Diabetes and Endocrinology, Westmead Hospital and University of Sydney, PO Box 533, Wentworthville NSW 2145, Australia; 9 Prince of Wales Hospital, High St, Randwick NSW 2031, Australia; 10 Acute Stroke Research, John Hunter Hospital and Hunter Medical Research Institute, Lookout Rd, New Lambton Heights NSW 2305, Australia; 11 Public Health Division, National Stroke Research Institute, Level 1, Neurosciences Building, Heidelberg Repatriation Hospital, Gate 10, 300 Waterdale Rd., Heidelberg Heights, Victoria 3081, Australia

## Abstract

**Background:**

Hyperglycaemia, fever, and swallowing dysfunction are poorly managed in the admission phase of acute stroke, and patient outcomes are compromised. Use of evidence-based guidelines could improve care but have not been effectively implemented. Our study aims to develop and trial an intervention based on multidisciplinary team-building to improve management of fever, hyperglycaemia, and swallowing dysfunction in patients following acute stroke.

**Methods and design:**

Metropolitan acute stroke units (ASUs) located in New South Wales, Australia will be stratified by service category (A or B) and, within strata, by baseline patient recruitment numbers (high or low) in this prospective, multicentre, single-blind, cluster randomised controlled trial (CRCT). ASUs then will be randomised independently to either intervention or control groups. ASUs allocated to the intervention group will receive: unit-based workshops to identify local barriers and enablers; a standardised core education program; evidence-based clinical treatment protocols; and ongoing engagement of local staff. Control group ASUs will receive only an abridged version of the *National Clinical Guidelines for Acute Stroke Management*. The following outcome measures will be collected at 90 days post-hospital admission: patient death, disability (modified Rankin Score); dependency (Barthel Index) and Health Status (SF-36). Additional measures include: performance of swallowing screening within 24 hours of admission; glycaemic control and temperature control.

**Discussion:**

This is a unique study of research transfer in acute stroke. Providing optimal inpatient care during the admission phase is essential if we are to combat the rising incidence of debilitating stroke. Our CRCT will also allow us to test interventions focussed on multidisciplinary ASU teams rather than individual disciplines, an imperative of modern hospital services.

**Trial Registration:**

Australia New Zealand Clinical Trial Registry (ANZCTR) No: ACTRN12608000563369

## Background

There are well recognised gaps in the implementation of best clinical practice in acute stroke care [1,2]. Important among these are the acute management of physiological variables known to influence stroke outcome. Elevation of blood glucose and body temperature in the early post-stroke period are associated with significantly worse stroke outcomes [3-8]. Management of swallowing dysfunction (dysphagia) also is crucial [9-11]. One of the greatest risks following stroke for a patient with a swallowing abnormality is aspiration which will lead to chest infections, aspiration pneumonia and death [12,13]. National guidelines affirm the importance of personnel on the clinical team specifically trained in swallowing screening as well as professional expertise in therapy and management [14]. Optimal management of these three clinical issues, namely fever, blood sugar, and swallowing are pivotal for favourable patient outcomes following stroke. All three have been identified as priorities for inpatient stroke management by Australia's peak body that sets standards in cerebrovascular disease, the National Stroke Foundation (NSF)[14]. Worryingly, while clinical practice guidelines recommend interventions to avoid and manage fever, elevated blood sugar, and swallowing, Australian data indicate that these factors are poorly managed [15].

New South Wales (NSW) has the highest number of acute stroke units (ASUs) of all states and territories in Australia [16]. Increased funding has been secured to promote best practice in ASUs, following government commitments to support evidence-based care. From 2002 to 2007, the number of ASUs has increased from seven to 23. Due to population distribution, all 23 are located in Sydney, Wollongong, and Newcastle [16], however more recently, initiatives to assist establishment of rural ASUs in NSW have been commenced [17]. In NSW, hospitals are classified into one of four categories (A, B, C, or D) based on criteria including the structure of stroke services, the processes of care available, and the clinical profile of patients (Table 1) [16]. Among key differences (Table 1), category A and B hospitals have access to more comprehensive acute-care services, such as on-site computerised tomography (CT) scanning and intensive care/high dependency beds. Category A hospitals also have on-site neurosurgery (Table 1). The majority of ASUs in NSW (n = 20) are classified as category A or B.

**Table 1:** National stroke unit program model [16]

**Components of care**	**Category A**	**Category B**	**Category C**	**Category D**
Immediate access to CT	Yes	Yes	Yes (within 24 hrs)	No
Access to high dependency unit	Yes	Yes	No	No
Onsite neurosurgery	Yes	No	No	No
Geographically located stroke unit	Yes	Yes	Yes (or a mobile stroke team with a care plan)	No

In an initiative to promote quality improvement in ASUs, the NSW Clinical Excellence Commission and the Royal Australasian College of Physicians initiated the Towards A Safer Culture (TASC) Clinical Support Systems Program [18]. TASC consists of an on-line, web-based data acquisition and feedback system for minimum and extended data sets. TASC embeds evidenced-based clinical practice with clinical quality improvement activities in NSW ASUs, providing clinicians with timely data about process and hospital outcome data for stroke patients.

Experts advise that efforts to assure evidence-based practice ought to themselves be based upon evidence [19,20]. Interventions must address barriers to guideline implementation [20,21]. Yet it is clear there is no one 'magic bullet' to assure evidence-based practice [22,23]. As a growing field of scientific inquiry, implementation research includes experimental designs in order to advance our understanding of what works to promote evidence-based practice, in what circumstances, and why [22,23]. Yet there has been little Australian research into guideline implementation. Further scientific study of barriers and intervention effectiveness within Australia has been advocated as a priority for implementation research for some time [24]. Certainly, too few rigorous evaluations have been conducted to examine the impact of better multi-disciplinary and inter-professional collaboration on patient care outcomes [25].

In our study, we will develop, implement, and rigorously evaluate a multidisciplinary team-building intervention in ASUs. Our intervention is designed to improve outcomes for patients admitted with acute stroke by better management of fever, hyperglycaemia, and swallowing dysfunction as recommended by evidence-based guidelines. This intervention will comprise replicable steps to identify local barriers and enablers, unit-based education, feedback, and ongoing proactive support. As we are focussing on **fe**ver, hyperglycaemia ('**s**ugar'), and **s**wallowing dysfunction, our intervention is known as the 'FeSS' intervention. Because the team-building intervention can only be delivered at the service level, we will randomise ASUs. As outcomes will be assessed at the patient level, we therefore have designed a cluster randomised controlled trial (CRCT)[26]. Recognising the emerging methodological interest in this design type, we have registered our trial  and, in this article, prospectively provide the research protocol. We do so also to promote technical developments in implementation research [22].

## Methods

### Investigators

The trial steering committee (SM, CL, JW, JG, RG, CD, SD, WC) has combined expertise in undertaking CRCTs, health service research, and nursing research, as well as content expertise in stroke management, clinical leadership, and adult education.

### Aims

To evaluate the impact on patient outcomes of our multidisciplinary team-building intervention designed specifically to improve evidence-based management of fever, hyperglycaemia, and swallowing dysfunction in patients following acute stroke. Specifically, we will test four primary hypotheses and three secondary hypotheses as follows:

### Hypotheses

That patients, admitted to ASUs randomised to receive the FeSS intervention will have, compared to patients treated in ASUs randomised to the control group:

#### Primary hypotheses

##### Patient outcomes

1. 12% lower death or disability at 90 days post-hospital admission (disability defined as Modified Rankin Score (mRS) ≥ 2)

2. 0.25 standard deviations lower mean disability (mRS) at 90-days post-hospital admission (0.5 units on mRS scale)

3. 0.25 standard deviations lower mean dependency score at 90-days post-hospital admission (as measured by the Barthel Index)

4. 0.25 standard deviations higher mean MCS and PCS SF-36 health status scores at 90-days post-hospital admission (2.5 units for PCS; 3.5 units for MCS).

#### Secondary hypotheses

##### Clinician behaviour change outcomes

1. Improved glycaemic control as measured by: 0.25 standard deviations lower mean finger-prick blood glucose level (BGLs) for the first 72 hours following admission (while finger-prick BGLs are not the 'gold standard' measurement method for blood glucose, they are currently routinely used for monitoring in clinical practice)

2. Improved temperature control as measured by: 0.25 standard deviations lower mean temperature readings for the first 72 hours following admission to the ASU

3. Improved management of swallowing dysfunction as measured by: 13% increase in the proportion of swallowing screening undertaken within the first 24 hours of admission to the ASU

In addition, in order to assess an overall measure of clinician compliance, we will compare between groups, the proportion of patients who meet the applicable clinical care elements (explained in depth below).

##### Participants: ASUs and their patients

Patients admitted to any of the consenting 20 category A and B ASUs in NSW will be eligible to participate in our CRCT. Medical directors and nurse unit managers (NUM) of all category A and B ASUs in NSW will be each sent a letter briefly outlining the study. Following this, CL and SM will meet face-to-face with the Medical Director and the NUM of each ASU to fully explain the study and obtain informed consent. To describe key commitments if agreeing to participate, CL and SM will inform these Medical Directors and Nurse Unit Managers that both control and intervention ASUs will receive information about evidence-based recommendations for the management of fever, hyperglycaemia, and swallowing dysfunction. They also will be informed that should their ASU be allocated to the intervention group, two workshops and two education sessions will be required to be held in their ASU in order to support evidence-based clinical treatment protocols for the management of fever, hyperglycaemia, and swallowing dysfunction.

The medical directors from those ASUs who agree to participate will be assigned to act as cluster guardians [27], signing a consent form for baseline data collection, randomisation to one of two groups (namely control or intervention), and implementation in their ASU of the FeSS intervention if allocated to the intervention group. If necessary, the cluster guardians will also consent to access by the researchers to the TASC clinical support system database for additional data for consenting patients. The project officer (SD) will archive ASU consent forms and assign study codes in order to maintain confidentiality.

#### Patient recruitment

##### Patient inclusion and exclusion criteria

To obtain baseline outcome and care data at the patient level, we will recruit a consecutive sample of English-speaking patients, aged >18 years, presenting within 48 hours of onset of symptoms who are given a clinical diagnosis of ischaemic stroke or intracerebral haemorrhage that is subsequently confirmed by CT imaging. These clinical criteria are specific and standard for stroke research. Patients will be excluded if they present to the ASU 48 hours or greater following onset of symptoms, have non-cerebrovascular causes of acute focal neurological deficits (seizure, hypoglycaemia, toxic or metabolic encephalopathies), sub-arachnoid haemorrhage, or acute and chronic subdural haemorrhage. Patients who require palliative care will not be approached.

All eligible patients will be approached by clinical research assistants (CRAs) identified in each ASU using a recruitment script. If an eligible patient agrees to participate in the study, they (or their family representative) will agree that researchers can contact them after 90 days of admission for a telephone interview; that researchers can access their medical records, and that TASC database can be accessed for their identified admissions data.

Our 90-day follow-up will comprise a computer-assisted telephone interview (CATI). One week prior to this CATI, a reminder letter will be mailed by the project officer (SD) to each participating patient. All CATIs will be undertaken by research interviewers blind to the study design and also to ASU group allocation [26,28]. These research interviewers all will have previous relevant experience and training in telephone administration of study measures. This CATI will include standard instruments as reported elsewhere in the section headed 'outcomes measures'.

#### ASU randomisation

**Figure 1:**
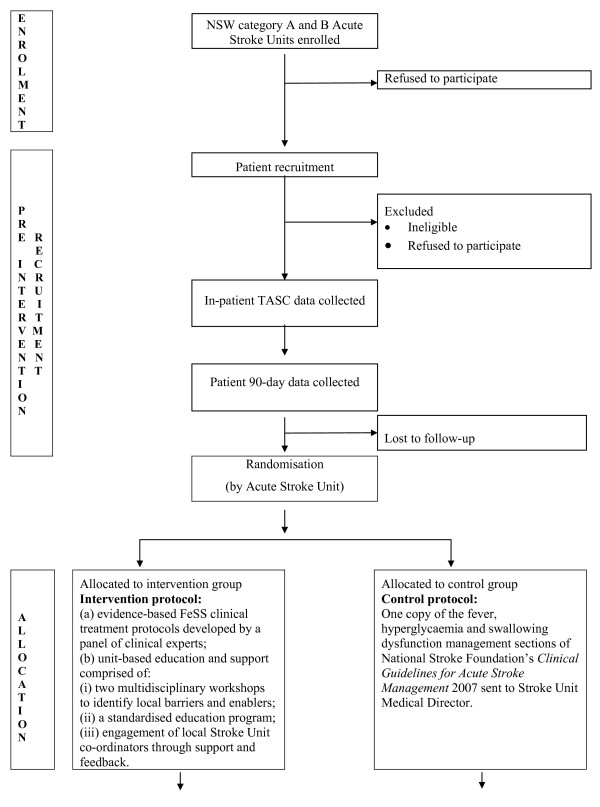
**CONSORT Flow diagram of the progress through the phases of the trial (part 1) **[26].

**Figure 2:**
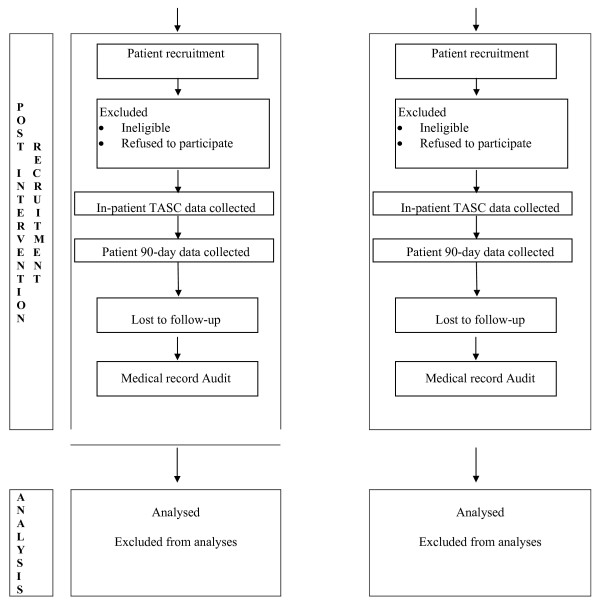
**CONSORT Flow diagram of the progress through the phases of the trial (part 2) **[26].

Once the baseline patient cohort 90-day outcome data have been collected, participating ASUs will be stratified and randomised. The project officer (SD) will first stratify ASUs according to their category classification (A or B) and then, by referring to absolute numbers of patients recruited at baseline, describe each as a 'high recruiter' or 'low recruiter'. Recruitment numbers will be included as a randomisation strata to maximise the chance of similar sample size in the intervention and control groups. Stratification details will be provided in a de-identified form to an independent statistician located offshore and not otherwise involved in the study for randomisation within strata, this will be generated using random number generating software[29]. Allocation will be based on clusters (ASUs) rather than individuals, and the sequence will be concealed until the intervention is assigned. Thus, generation of the allocation sequence and assigning of ASUs to either intervention or control group will be undertaken by the offshore independent statistician. To strengthen our methodological rigour, personnel who recruit patients (CRAs), research interviewers who undertake the CATIs, and the offshore statistician who undertakes randomisation all will be independent and also blinded to all other components of the study design. A flow diagram further outlining the trial design is shown in Figures 1 and 2.

After randomisation, the FeSS intervention will be implemented at those ASUs randomised to the intervention group. Following a minimum period of three months to allow the FeSS intervention to become an integral part of usual clinical practice in the intervention ASUs, a subsequent sample of patients will be recruited from all ASUs to provide post-intervention outcome data. These data will be collected using identical tools and methods to those used to collect baseline data. For the purpose only of temporal equity, each intervention ASU will be paired with a control ASU from the same category to calibrate the timing of post-intervention data collection.

#### The FeSS intervention

As has been concluded elsewhere[30], there is not yet one cohesive theoretical framework for knowledge transfer in clinical practice improvement. Indeed, over 60 potential theories or models can be used [31]. A certain theoretical pluralism has been recommended [30]. Our practical approach has nonetheless drawn heavily from the implementation literature to incorporate promising strategies that have, in other settings, improved the provision of evidence-based clinical care. We will deliberately focus on multidisciplinary team-building. Hence, we have incorporated the following: early and widespread involvement of staff using formal facilitation methods [32,33]; high quality training materials with timely on-the-job training [32,34-36]; team-based training (as opposed to individual training) [37]; encouraging adaptation of the intervention to the local context [34,38,39]; and involvement of staff in evaluating the success of local adoption of intervention [38].

In addition, our intervention was informed by the systematic review undertaken by Grimshaw *et al*. [40] and, as such, includes use of reminders, educational outreach, and dissemination of educational materials.

To prevent contamination before project completion, broad components only of the FeSS intervention will be described in this study protocol. First, the FeSS intervention will provide evidence-based clinical treatment protocols for the management of fever, hyperglycaemia, and swallowing dysfunction to ASUs allocated to the intervention group. These protocols will be developed by three panels of clinical experts (one for each clinical focus). Following development of the FeSS clinical treatment protocols, we will conduct two multidisciplinary on-site workshops. The first workshop will target senior clinical ASU members (medical director, nurse unit manager, stroke unit co-ordinator (clinical nurse consultant), stroke fellow/registrar, director of speech pathology) in order to identify barriers within the ASU and also in the broader hospital context. At this time, we will also identify and engage key champions in each ASU, such as the nurse educator and speech pathologist. Preliminary recommendations for the process of local implementation of the FeSS clinical treatment protocols also will be discussed at this first workshop. Any necessary local modifications to the FeSS clinical treatment protocols will be discussed and undertaken by the researchers. At the second workshop, the FeSS clinical treatment protocols (with requested local modifications where applicable) will be presented to a multidisciplinary audience comprising bedside nurses and the ASU speech pathologists to identify any additional barriers within the ASU. Following this, further revisions to the FeSS clinical treatment protocols will be made where recommended. In order to assure integrity and consistency of the FeSS clinical treatment protocols at all intervention sites, the three panels of clinical experts who develop the clinical treatment protocols will predetermine the 'minimum clinical care elements, *i.e*., those elements that will not be permitted to be altered at local sites (specifically, target BGLs and target temperatures). Three of the authors (SM, CL and SD) will convene these workshops to ensure consistency in delivery.

In those units allocated to the intervention group, the project officer (SD) also will deliver unit-based education and support. To ensure complete coverage of clinical personnel, each ASU will be offered two identical education sessions to be scheduled at different times. The aim of these sessions is to educate nurses about the clinical treatment protocols. A standardised PowerPoint presentation and accompanying handouts will be made available for further use to the nurse and speech pathologist responsible for education of nurses on each ASU as identified at the first multi-disciplinary workshop. The nurse and speech pathologist will conduct further education events as required to ensure all nursing staff, including night staff, are educated about the elements of the FeSS clinical treatment protocols. Finally, longitudinal engagement through support and feedback will be provided by the project officer (SD) on an ongoing basis for the duration of the intervention. The project officer (SD) and SM will establish personal links with the stroke unit co-ordinator at all ASUs and others identified as key champions at the first multidisciplinary workshop. Thus, it can be seen that our intervention is both organisational, inter-professional (involving all team professionals), and patient-based (offering and refining clinical treatment protocols).

Clinical nursing staff at the intervention ASUs will be able to undertake optional audits in their own ASU if they wish to monitor local implementation of the FeSS clinical treatment protocols. To support this optional activity, we will provide audit tools but will not be supporting data collection or analysis. This element will likely encourage clinical ownership for implementation at the local level [38].

Control group ASUs will only receive an abridged version of the latest NSF Guidelines for Acute Stroke Management [14]. While these guidelines usefully outline recommendations relevant to the management of fever, hyperglycaemia, and swallowing dysfunction, there will be no additional effort to disseminate or implement them in these units [41].

### Outcome measures

#### Patient outcome measures

1. Death or disability at 90 days post-hospital admission. Disability will be defined as a mRS of ≥ 2 [42,43]. Our CRAs at participating ASUs will be asked to inform us when patients enrolled in the study die while in hospital. Our letter to consenting patients one week prior to the CATI will enable relatives to contact the researchers to inform us of any patient death following discharge.

2. Level of disability at 90 days post-admission using the modified Rankin Score (mRS) [42,43], a six point measuring independence rather than performance of specific tasks. The scale ranges from zero to six, where zero corresponds with no symptoms, five corresponds to severe disability, and six corresponds with death. Disability will be defined as a mRS of ≥ 2 [42].

3. Level of dependency 90 days post-hospital admission using the Barthel Index (BI) [44]. The BI measures patient performance in 10 activities of daily life. The items are divided into groups that relate to self-care (feeding, grooming, bathing, dressing, bowel and bladder care, and toilet use) and a group related to mobility (ambulation, transfers, and stair climbing). The maximal score is 100 if five-point increments are used indicating the patient is fully independent in physical functioning. The lowest score is zero, representing a totally dependent bedridden state.

4. Health status 90 days post-hospital admission using the Medical Outcomes Study Short Form 36 Health Survey Questionnaire (SF-36) [45]. The SF-36 includes a single 'health transition rating' and scores eight health domains which are aggregated to form the Physical Component Score (PCS) and the Mental Component Score (MCS). Higher mean scores reflect better states of health and wellbeing [45]. This measure of self-perceived general health status is particularly sensitive to change between one to three months following stroke [46].

#### Behaviour change outcome measures

1. Improved glycaemic control as measured by: mean finger-prick BGL readings for first 72 hours following admission to ASU.

2. Improved temperature control as measured by: mean temperature readings for the first 72 hours following admission to the ASU.

3. Improved management of swallowing dysfunction as measured by: swallowing screen undertaken within the first 24 hours of admission to the ASU.

All outcome measures listed above apply at the level of the patient and will be clustered at the ASU level. As previously stated, we will compare between groups, the proportion of patients who meet the applicable clinical care elements (Note: not all elements will apply to all patients) to obtain an overall measure of clinician compliance for each ASU.

#### Other Clinical Measures

In addition, the following clinical measures will be used to describe and compare the groups and considered as potential confounders:

1. Stroke subtype: Using the Oxfordshire Community Stroke Project (OCSP) Classification [47], a four item scale that classifies strokes using explicit criteria as either lacunar infarcts, total anterior circulation infarcts, partial anterior circulation infarcts, or posterior circulation infarcts.

2. Stroke severity: Using the Scandinavian Stroke Scale (SSS)[48], a measure of stroke severity involving assessment of the following parameters: consciousness, eye movement, motor power – arm, hand, leg, orientation, speech, facial palsy and gait to be measured on admission. Score range from zero to 58; the Los Angeles Motor Scale (LAMS), a motor deficit score ranging from zero (least affected) to ten (most affected) for bilateral weakness and zero to five in patients with unilateral weakness [49,50].

3. level of pre-morbid disability using the mRS [42,43]

4. demographic variables: age, sex, date of hospital admission, and length of stay

For missing data, patient clinical data will be obtained from the TASC database. Patients themselves will already have agreed to allow access to these data as part of the study consent. For hospitals that do not collect TASC data, stroke severity, stroke sub-type, level of pre-morbid mRS, and demographic variables will be prospectively manually collected from patient medical records by CRAs at each participating site following patient recruitment.

#### Professional behaviour change outcome measures

Changes in professional care also will be determined. Data will be obtained by retrospective medical record audit undertaken by independent research assistants (IRAs) blind to group allocation. A data dictionary will be developed and all research assistants will undergo training; inter-rater reliability testing will be undertaken (see 'behaviour change outcome measures' section for list of data to be collected).

### Blinding

Both the medical director and NUM of all consenting ASUs will be aware that our study is examining the effect of an intervention to manage fever, hyperglycaemia and swallowing dysfunction following acute stroke. Furthermore, as control ASUs receive a minimum intervention, medical directors and NUMs from ASUs subsequently randomised to the control group may be able to deduce their group allocation because no workshops are being organised. However, all senior clinical members of control group ASUs remain blind to the exact nature of the intervention as described above.

CRAs recruiting patients will be blind at baseline to ASU group allocation. While some CRAs may infer group allocation at post-intervention data collection, they are responsible only for patient recruitment and not collection of outcome data *per se*. Patients will be blinded to group allocation. Data entry will be undertaken by the CATI research assistants blind to group allocation.

### Data Analyses

Blinded outcome assessment will be undertaken for all analyses of primary and secondary outcome measures. Data will be analysed using Stata [51]. Intention-to-treat analysis will applied [26].

To examine potential response bias, demographic characteristics for eligible consenting and non-consenting patients will be compared using the chi-square test for categorical variables (sex, stroke sub-type, and stroke severity) and the t-test (or a non-parametric equivalent) for the continuous variable of age.

For all patient-related outcome analyses, we will use cluster-specific methods. Dichotomous outcomes (death or disability [mRS ≥2] at 90 days; swallowing screen within 24 hours of admission) will be compared between intervention and control groups using the chi-square test. Continuous outcomes – level of disability (mRS score), dependency (Barthel Index), health status (MCS and PCS of SF-36), improved glycaemic control, and improved temperature control – will be compared between the two intervention groups using the t-test. The survey (svy) commands in Stata will be used to adjust for clustering of patients within ASUs. Multilevel modelling (logistic or linear as appropriate) will be used to compare primary outcomes – death or disability (mRS or ≥2) at 90 days, level of disability (mRS score), dependency (Barthel Index), and health status (MCS and PCS of SF-36) – between groups while adjusting for potential confounders or effect modifiers and for the cluster study design. These analyses will be undertaken in the Stata statistical package [51].

### Sample Size

TASC data from January 2003 to May 2005 demonstrated that 35% of patients had a mRS ≥2 at hospital discharge, and the mean hospital discharge mRS was 2, with a standard deviation of 2. A sample of 250 per group would allow detection of a difference between groups of 12% (35% versus 23%) for the proportion of patients with death or disability (≥2 on the mRS) and a clinically meaningful difference in mean mRS of 0.5 (from 2 to 1.5, equivalent to a 25% change in mean score) with 80% power and a 5% (two-sided) significance level. This sample would also allow detection of differences between groups of at least 13% for binary outcomes and one-quarter of a standard deviation for continuous outcomes, with 80% power and a 5% (two-sided) significance level. Assuming a loss to follow-up of 10%, an effective sample size of 280 participants per group is required to be recruited. These calculations assume independent observations. We devised a table to demonstrate statistical power according to various defensible estimates of intra-cluster correlation co-efficients (ICCs) for these two patient outcomes (Table 2). Estimated ICCs range from 0.01 to 0.03 [52]. We anticipate a design effect of 1.85, thus aim to recruit 520 patients per group (1,040 in total).

**Table 2:** Effective sample size, assuming different magnitudes of intracluster correlation (ICC)^

**ICC**	**Design Effect**	**Number of patients per group**	**Total number of patients required**
0	1	280	560
0.01	1.4	400	800
0.015	1.85	520	1040
0.02	2.6	730	1460
0.03	4.4	1230	2460

^ 80% power at alpha = 0.05; nine ACU's per group; adjusted for loss to follow-up of 10%

### Ethical Approval

This CRCT has been approved by the National Human Research Ethics Committee of the Australian Catholic University and the relevant Human Research Ethics Committees of all participating hospitals. Use of TASC data has been approved by the NSW Department of Health Ethics Committee.

## Discussion

Fever, hyperglycaemia, and swallowing dysfunction are recognised to be associated with unfavourable clinical outcomes post-stroke, and all international stroke care guidelines recommend prompt assessment and treatment of these factors [14,53,54]. To our knowledge, this also will be the first intervention research in stroke to implement and to determine the impact of a standardised, multidisciplinary team-building intervention to manage these three common stroke co-morbidities and complications. We have adopted a CRCT design to rigorously evaluate our intervention in order to avoid logistical and methodological issues that arise when conducting health services research [55]. This research design will minimise and at best, eliminate, contamination. Our trial is testing an enhanced organised acute stroke care model where there may be additional patient outcome benefit above that accrued from organised stroke care alone. Recent results from the National Stroke Audit demonstrate gaps in stroke best clinical practice[1]. Our trial will address such clinical practice gaps and, as such, is highly significant both within Australia and internationally.

## Competing interests

The authors declare that they have no competing interests.

## Authors' contributions

SM, CL, JW, JG, and CD conceived and developed the study, drafted the study protocol and secured funding. SD coordinates the ongoing study and contributed to research materials. RG, CD, CQ, ME and DC provided input on the design. WC, CQ, and ME have contributed to aspects of the protocol. SM, CL, CQ, WC, ME, and DC finalised components of the intervention. All authors have read and approved the final manuscript, and take public responsibility for its content
